# Supplementary material for: Coping with chronic periprosthetic joint infection after failed revision of total knee and hip arthroplasty: a qualitative study on patient’s experiences in treatment and healing
Source: PLoS One. 2025 Mar 12;20(3):e0319509. doi: 10.1371/journal.pone.0319509 (PMC11902299; doi:10.1371/journal.pone.0319509)
Supplement: S1 File — (DOCX) [file pone.0319509.s001.docx]

***Note: As the interviews were conducted in German, these are the translated versions.***

**Interview guide for first Interviews**

Introduction:

My name is X. I am a psychologist and medical doctor, and I am leading the research project in which we are looking at different experiences of people with PJI and how they have experienced their illness to date. I am interested in the impact of the complications on your everyday live, how personally perceive them and what helped in order to deal with the challenges.

Before the interview:

Do you have any questions about the research project?

Do I have your permission to record the interview?

1. To begin with, I would like to ask you to describe your current situation with your endoprosthesis.
   1. Can you please give me an example of … (aiming at elaboration of specific situations that were described)?
2. What currently helps you to deal with this situation?
   1. Examples …
3. How did it come about that you find yourself in the current situation? (AT)
   1. What were the key moments that led to your current situation?
      1. Examples …
   2. What helped you to overcome the situation (A or B)?
      1. Examples …
   3. What were your considerations and thoughts in these situations (A or B)?
      1. Examples …
4. What helped you, what supported you in these situations you just described to me?
   1. Examples …
5. What are your expectations for the future?
   1. Examples …
6. What are your wishes for the future?
   1. Examples …
7. What needs to happen for you to be satisfied with your situation?
   1. Examples …
8. What could support you in the future?
   1. Examples …
9. Is there a question I have not asked you?
10. Is there a question a treating physician should have asked you?
11. Have you reached out to a psychologist/psychiatrist since the onset of the PJI?
12. Would you be interested in participating in a second interview six month from now?

**Interview guide for follow-up interviews**

Reaching out to patients on telephone:

Hello, my name is X and I am calling from the department XXX. We already completed an interview together a few months ago. We have now started a second series of interviews and I wanted to ask you if you would still be interested in taking part in a second interview? The second interview usually lasts 30 minutes.

I can offer you that we can either do this in person at our department or at your home or we undertake the whole interview on the phone?

Introduction:

Thank you again for taking the time for the second interview. Today I want to address my questions towards the current state you’re in regarding your situation with your endoprosthesis and elaborate if and what has changed since our last interview.

Before I ask my questions, do you have any questions you would like to address to me?

Do I have the permission to record the interview?

1. When you think back to the first interview, what do you remember from that time?
2. Have there been any changes in your life regarding your endoprosthesis since the last interview?
   1. Examples …
3. Can you please describe me what your current situation looks like?
   1. What has improved?
      1. Examples …
   2. What has worsened?
      1. Examples …
4. What currently helps you to deal with this situation?
5. What could help you in your current situation?
   1. Examples …
6. What are your current expectations for the future?
   1. Examples …
7. If you were to give a tip to someone living with an infected endoprosthesis, what would it be?
8. Is there a question I have not asked you?
9. Is there a question you would like to ask me?
10. May I have the permission to contact you again in the future for another interview?
11. I would like to offer you my email address and telephone number in case you have any further questions.
