# Supplementary figures and images for: Coping with chronic periprosthetic joint infection after failed revision of total knee and hip arthroplasty: a qualitative study on patient’s experiences in treatment and healing
Source: PLoS One. 2025 Mar 12;20(3):e0319509. doi: 10.1371/journal.pone.0319509 (PMC11902299; doi:10.1371/journal.pone.0319509)

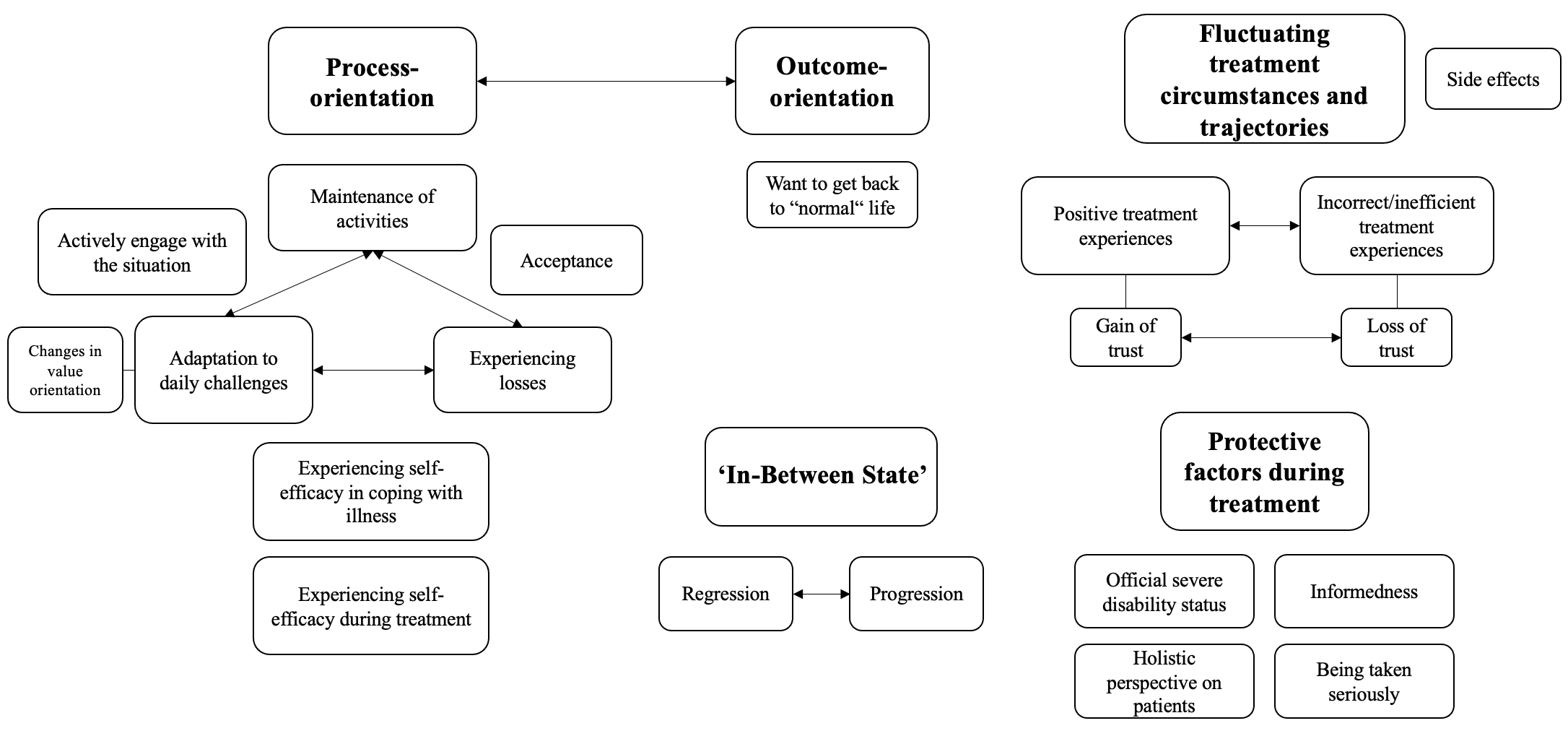

Supplement: S2 Fig — (TIF) [file pone.0319509.s002.tif]

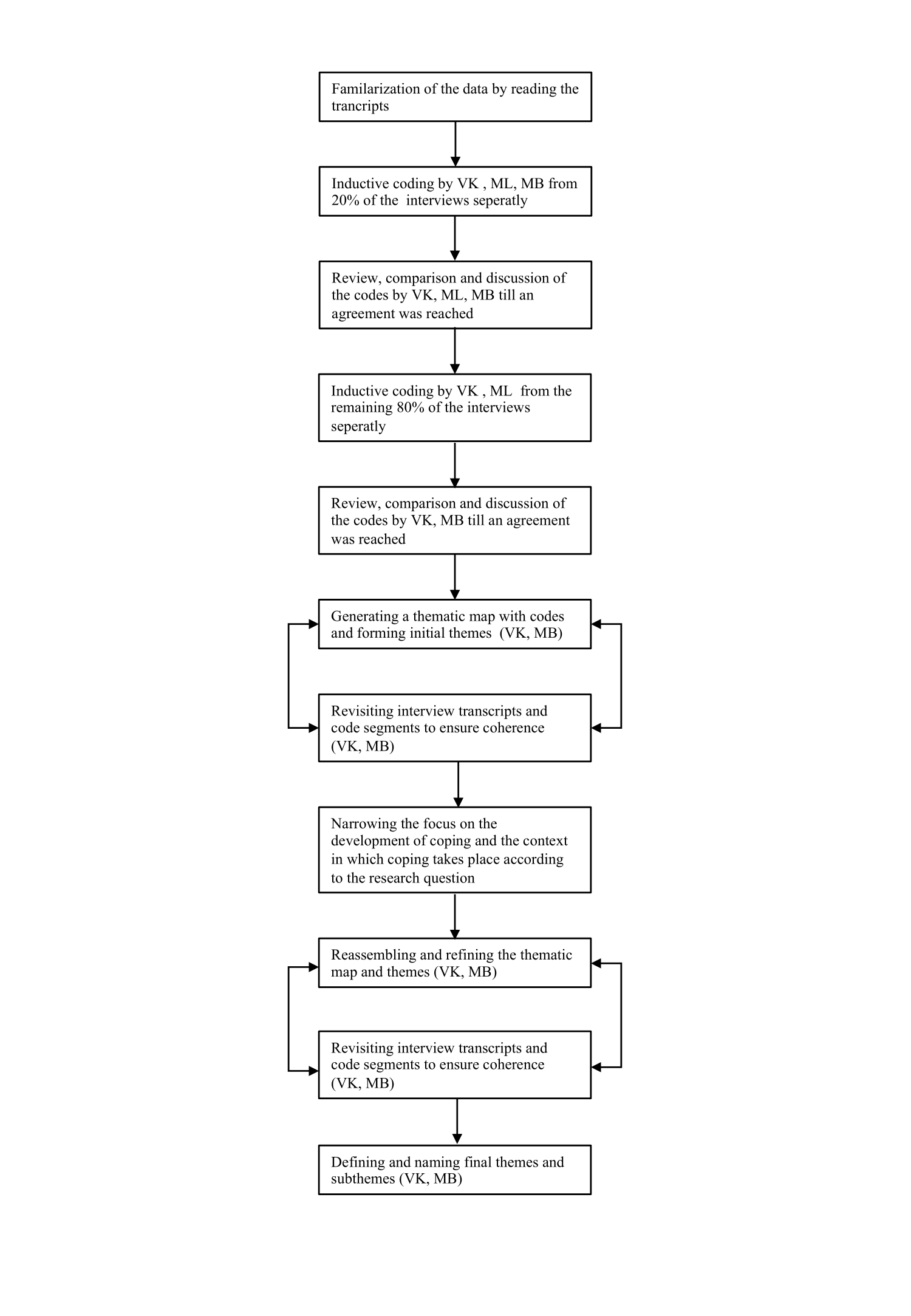

Supplement: S4 Fig — (TIF) [file pone.0319509.s004.tif]
