# Supplementary material for: Coping with chronic periprosthetic joint infection after failed revision of total knee and hip arthroplasty: a qualitative study on patient’s experiences in treatment and healing
Source: PLoS One. 2025 Mar 12;20(3):e0319509. doi: 10.1371/journal.pone.0319509 (PMC11902299; doi:10.1371/journal.pone.0319509)
Supplement: S3 Table — (DOCX) [file pone.0319509.s003.docx]

| **Domain 1: Research team and reflexivity** |  |
| --- | --- |
| **Personal Characteristics** |  |
| Interviewer/facilitator | Correspondant author (VK) conducted all interviews |
| Credentials | VK = Dr. med. Univ & M. Sc.  MB = M. Sc.  SM = Dr. med.  CP = Prof. Dr. med.  AT = Prof. Dr. med.  ML = Dr. med. Univ & M. Sc. |
| Occupation | VK = physician, psychologist M.Sc, doctoral programs for Dr. rer. medic. and Dr. med.  MB = clinical psychologist M.Sc, psychotherapist in training, doctoral program for Dr. rer. medic.  SM = Surgeon specialized on septic surgery  CP = Orthopedic surgeon and department head for orthopedic surgery  AT = Senior physician specialized on infectious diseases and department head of the septic surgery ward  ML = physician, psychologist M.Sc., PhD student |
| Gender | VK, SM, AT, CP, ML are male; MB is female |
| Experience and training | VK is an experienced researcher with twelve Pub Med listed articles where he primarily focused on the diagnostics of PJI. He has a solid background in qualitative research due to his doctoral training and his M. Sc. in Psychology. He joined the research team of AT for PJI for 1,5 years and is well versed in the treatment regimens of PJI. |
| **Relationship with participants** |  |
| Relationship established | No relationship was established prior to study commencement. |
| Participant knowledge of the interviewer | The interviewees knew that the research project was part of the doctoral studies of VK and that he was trained both in psychology and medicine. Upon recruiting, the interviewees were informed that the questions would focus on their personal experiences regarding treatment and healing considering their whole treatment journey with the PJI. |
| Interviewer characteristics | Since this paper is part of VK doctoral studies he has a bias towards successful publishing. Further, VK worked for 1,5 years on the infectious ward and was thus exposed to a multitude of PJI cases which biased him to clearly state the need for psychological aid. His training in medicine and psychology influenced him to choose a topic for his doctoral studies that bridges both disciplines and further unites his prior publication interest in PJI diagnostics. VK was not involved in the treatment of patients on the ward where patients were placed so that interviewees did not get the impression that the outcome of the interview might affect their treatment. Interviewees knew in advance to the interview that VK has a training in psychology which might have influenced them to overstate their mental burden. |
| **Domain 2: Study design** |  |
| **Theoretical framework** |  |
| Methodological orientation and theory | Thematic analysis was used as a relativistic, inductively oriented analysis, grounded in an experiential framework. |
| **Participant selection** |  |
| Sampling | Purposive sampling of chronic cases of PJI |
| Method of approach | Face-to-face in the outpatient clinic |
| Sample size | 18 |
| Non-participation | Participation refused by patients: 2.  Drop-outs (1. Interview): 2 (due to severe emotional distress, the interview was aborted)  Drop-outs (2. interview): 3 (1 reached critical health status, 2 were unable to reach for the follow-up interview) |
| **Setting** |  |
| Setting of data collection | 1. Interview: all were conducted face-to-face at the outpatient clinic in a private room  2. Interview: 12 via telephone, 3 face-to-face at patients’ homes |
| Presence of non-participant | Since many patients were in company of relatives they were given the choice of being interviewed alone or in presence of the accompanying person.  In the case of Berta (both 1. and 2. Interview) and Frida (only 1. Interview) the husbands were present upon request. Both (Berta & Frida) felt that the disease is an immanent matter of their relationship and therefore wanted to include their husbands. During the interview of Annette her niece was present upon request. |
| Description of sample | 10 female and 8 male patients, age ranged from 55 to 92 |
| **Data collection** |  |
| Interview guide | Questions were provided by the author; interview guide was pilot tested and adapted after three trial interviews |
| Repeat interviews | 1 follow-up interview was conducted six months after the first interview |
| Audio/visual recording | Audio recording was used to collect the data |
| Field notes | Field notes were taken after each interview and were shared with the coders MB & ML |
| Duration | On average 52 minutes for the first interview and 28 minutes for the follow-up interview |
| Data saturation | Thematic saturation was achieved and determined the sample size |
| Transcripts returned | No |
| **Domain 3: analysis and findings** |  |
| **Data analysis** |  |
| Number of data coders | 20% of the interviews (Three 1. Interviews and three 2. Interviews) were separately coded by VK, MB and ML and discussed.  VK and MB coded the rest of the interviews separately and discussed the codes afterwards. |
| Description of the coding tree | A coding tree is attached in the supplement material. |
| Derivation of themes | Themes were inductively derived from the data. They were not predefined prior to the analysis. |
| Software | MAXQDA24 for analysis and f4transkript for transcription |
| Participant checking | No |
| **Reporting** |  |
| Quotations presented | Quotations were presented to illustrate both major and minor themes. Each quotation was linked with the pseudonym of the interviewee. |
| Data and findings consistent | Yes. We see a clear consistency between the data and findings since we strictly analyzed the data regarding the initially posed research question. |
| Clarity of major themes | Yes. The two major themes are clearly outlined and distinct from each other. One major theme focuses on the coping and the other on the context in which this coping was developed. |
| Clarity of minor themes | Yes. The three subthemes of the first major theme (process-oriented coping) each represent one distinct aspect. Each subtheme (Adaptation to daily challenges and losses; Active expectation management; Self-efficacy as a key resource for navigating treatment and healing) is made of two distinct facets that are than elaborated further.  The second major theme (Fluctuating treatment circumstances and trajectories) entails three minor themes (Uncertainty and unpredictability; “In-between”-state; unstable patient-physician relationships) that elaborate the experiences within the context of a complex PJI. The minor theme of unstable patient-physician relationships further entails five facets (ineffective treatment, loss and gain of trust, holistic perspective, not being taken seriously, informedness) that outline aspects of these relationships. |
